# Cut‐Enabled Mechanical Metamaterials for Multimodal and Reprogrammable Static Nonreciprocity

**DOI:** 10.1002/advs.202503455

**Published:** 2025-08-20

**Authors:** Jinhao Zhang, Shuo Zhang, Xiao Zhou, Yueqi He, Fengwen Wang, Dianlong Yu, Yu Jiang, Mi Xiao, Xin Fang

**Affiliations:** ^1^ National Key Laboratory of Equipment State Sensing and Smart Support College of Intelligence Science and Technology National University of Defense Technology Changsha 410073 China; ^2^ State Key Laboratory of Bioinspired Interfacial Materials Science Bioinspired Science Innovation Center Hangzhou International Innovation Institute of Beihang University Hangzhou 311115 China; ^3^ Department of Civil and Mechanical Engineering Technical University of Denmark Koppels Allé Building 404 Kongens Lyngby 2800 Denmark; ^4^ State Key Laboratory of Intelligent Manufacturing Equipment and Technology Huazhong University of Science and Technology Wuhan 430074 China

**Keywords:** mechanical metamaterial, multimodal nonreciprocity, reprogrammable nonreciprocity

## Abstract

Static nonreciprocity offers distinct outputs when switching the positions of action and reaction, which is of great interest for designing mechanical logic elements or soft robots. Existing mechanical metamaterials can present specific static nonreciprocal responses, but it remains challenging to obtain multiple and reprogrammable static nonreciprocal modes in a single microstructural topology. Here, a design method of cellular metamaterials is demonstrated via leaving cuts inside metacells, whose contact nonlinearity in the single metacell can offer orthogonal, uniaxial, shear (displacement and Poynting effect) static nonreciprocal modes. A framework using constitutive tensors is established to describe the multi‐modal nonreciprocal behaviors. Moreover, the static nonreciprocal responses of the metamaterial array are programmable via encoding (retaining or constraining) the positions of cuts. This work offers a pathway to synthesize multiple nonreciprocal modes and control the nonreciprocal responses, enhancing the functionality of metamaterials.

## Graphical abstract

Cut‐enabled mechanical metamaterials are designed to overcome the challenge of obtaining multiple and reprogrammable static nonreciprocal modes in a single microstructural topology. It offers a pathway to synthesize multiple nonreciprocal modes and control the nonreciprocal responses, enhancing the functionality of metamaterials.

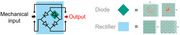

## 1 Introduction

The principle of reciprocity,^[^ ^1^, ^2^ ^]^ is general in linear physics.^[^ ^3^ ^]^ However, breaking reciprocity allows for the diode effects when switching the action and reaction positions, opening great potential for controlling material deformation and structural responses.^[^ ^4^, ^5^, ^6^ ^]^ Dynamic nonreciprocity enables unidirectional propagation,^[^ ^7^, ^8^, ^9^ ^]^ of electromagnetics waves^,[^ ^10^, ^11^, ^12^ ^]^ elastic waves,^[^ ^13^, ^14^ ^]^ and sound waves,^[^ ^15^, ^16^ ^]^ which have been used to design functional devices for energy switching and rectification, signal processing, vibration isolation, and shock protection.^[^ ^17^, ^18^, ^19^, ^20^, ^21^ ^]^

Static nonreciprocity in mechanical systems,^[^ ^22^, ^23^, ^24^, ^25^, ^26^, ^27^ ^]^ exhibits a direction‐dependent relationship between force and deformation,^[^ ^28^ ^]^ offering opportunities to design systems that support motion control,^[^ ^29^ ^]^ energy conversion,^[^ ^29^ ^]^ and biological manipulation.^[^ ^30^ ^]^ Recent advances in rationally designed metamaterials and structures,^[^ ^31^, ^32^, ^33^, ^34^, ^35^, ^36^ ^]^ have facilitated the realization of different static nonreciprocal modes.^[^ ^28^, ^29^, ^30^, ^37^, ^38^, ^39^ ^]^ For example, the fishbone‐like metamaterial can present a uniaxial nonreciprocal mode,^[^ ^28^ ^]^; the composite material embedded nanofillers exhibits a shear nonreciprocal mode.^[^ ^30^ ^]^

As well known, in the 2D space, one can use 9 elastic constants to describe material properties. This means the nonreciprocal behaviors between the coupling from different loading directions can be multimodal. However, though static nonreciprocity has attracted great attention, it remains challenging to obtain multiple static nonreciprocal modes in a single microstructural topology. Moreover, many strategies have been proposed for achieving reprogrammable responses,^[^ ^40^, ^41^, ^42^ ^]^ including multiple stable states,^[^ ^34^, ^43^ ^]^ controllable local tilting orientations,^[^ ^44^ ^]^ tunable shape,^[^ ^45^, ^46^ ^]^ and reprogrammable stiffness,^[^ ^47^, ^48^ ^]^ but reprogrammable nonreciprocal responses remain to be explored. Beyond the design, establishing a theoretical framework for understanding the mechanisms underlying nonreciprocity is also significant.

Here, we design the nonreciprocal metamaterials via controlling the cuts inside the metacell. Such same microstructural cell can present nonreciprocal modes in orthogonal, uniaxial, and shear loading cases. These nonreciprocal modes are characterized by different pairs of constitutive tensors. Furthermore, the static nonreciprocal behaviors can be reconfigured via introducing external constraints to cuts. Several potential applications of cut‐enabled nonreciprocal metamaterials are demonstrated in experiments.

## 2 Constitutive Asymmetry Induced by Static Nonreciprocity

**Figure 1:**
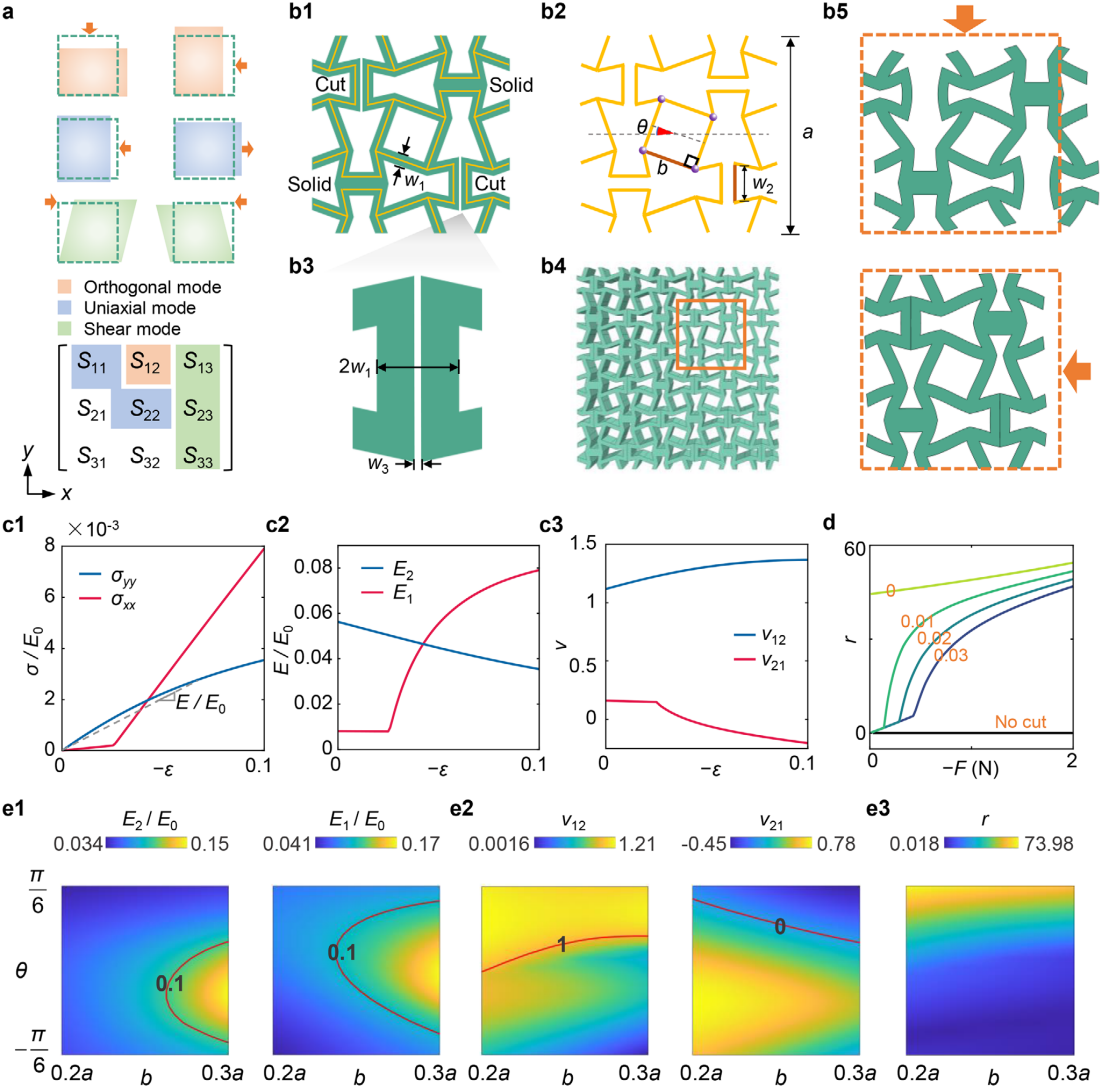
Deformation modes of different constitutive tensors and design framework of cut‐enabled mechanical metamaterials. a) Orthogonal, uniaxial, and shear modes and corresponding constitutive tensors (Note S1, Supporting Information). *S_ij_* is a compliance tensor. b1, b2, b3, b4) unit cell, microstructural topology, local cut, and structure in the design framework. b5) Simulations of a cell under the compressive strain of 10%, where *w*_1_ = 0.06*a*, *w*_2_ = 0.2*a*, *w*_3_ = 0.02*a*, *b* = 0.28*a*, and *θ* = *π*/9. c1, c2, c3) Effective stress and strain, secant elastic moduli, and secant Poisson's ratios of the deformed cell in Figure> 1b5. d) Constitutive asymmetry of the metamaterial with different *w*_3_. e1, e2, e3) Secant elastic moduli, secant Poisson's ratios, and constitutive asymmetry evaluated under small strains of 0.1%. *w*_3_ = 0.0002*a*, but a zero initial distance between the surfaces of the predefined cuts is set in simulations.

In classical elastic media, the constitutive relation between strain and stress is determined by the elastic compliance matrix *S*. In the compliance matrix, *S*_11_ (or *S*_22_) connects the strain and stress in a uniaxial direction; *S*_12_ (or *S*_21_) describes the coupling between two orthogonal axes; and *S*_33_ (or *S*_13_, *S*_23_) corresponds to shear deformation, as shown in **Figure** **1**a (Note S1, Supporting Information). The well‐known symmetry, *S_ij_* = *S_ji_*, stems from the principle of static reciprocity. For example, when applying a normal force *F*_1_ (or *F*_2_) in one axis and obtaining the output displacement *u*_1→2_ (or *u*_2→1_) in its orthogonal axis, the static reciprocity writes *F*_1_ *u*_2→1_ = *F*_2_ *u*_1→2_, and thus we yield constitutive symmetry *S*_12_ = *S*_21_ in two axes (Note S2, Supporting Information). In contrast, the system satisfying *F*_1_ *u*_2→1_ ≠ *F*_2_ *u*_1→2_ shows the constitutive nonreciprocity, *S*_12_ ≠ *S*_21_. We formulate the deviation between *S*_12_ and *S*_21_ to quantify the degree of static nonreciprocity under this pair of loads:

(1) $$r=\left|\left(S_{12}-S_{21}\right)/S_{12}^{*}\right|$$

where $S_{12}^{*}=S_{21}^{*}$ denotes the linear elastic constant of the metamaterial's primary material. Static nonreciprocity emerges when *r*≠0. The larger *r* manifests stronger static nonreciprocity.

## 3 Results and Discussion

### 3.1 Design Methods of Nonreciprocal Cellular Metamaterial With Cuts

We design 2D cellular metamaterials with preserved cuts in the periodic cell (Figure 1b). Its periodic cell in Figure 1b1 has a web‐like square configuration, and each wall has the same thickness *w*_1_ before cutting. The lattice constant is *a*. Two vertical cuts are left in every unit cell, with a tiny clearance inside the cut. The height and width of the cuts are *w*_2_ and *w*_3_, respectively. The arm length and rotation angle of the square are *b* and *θ*, respectively (Figure 1b2). The topology has *C*_4_ rotation symmetry and anti‐tetra‐chirality (Figure S1, Supporting Information), which also provides negative Poisson's ratios via local rotations under uniaxial loads.^[^ ^49^ ^]^ Specially, in the designed metamaterials, the connectivity of arms in the topology is changed by the solids between arms, breaking *C*_4_ rotation symmetry (Note S3, Supporting Information). The cuts provide favorable geometrical conditions for contact between arms.

We adopt the finite element method (FEM) to calculate the deformation modes with periodic boundary conditions (Note S4, Supporting Information). The clearance at cuts becomes larger when applying the longitudinal compression force from the *y*‐axis due to the local effect of positive Poisson's ratio near the cut; while the two walls of the cuts contact under the compression applied on the *x*‐axis, as shown in Figure 1b5. The contact causes a sharp increase in structural stiffness in the *x*‐axis, as shown in Figure 1c1 and c2, and the mutation also emerges in Poisson's ratios *v*_21_ (Figure 1c3).

**Figure 2:**
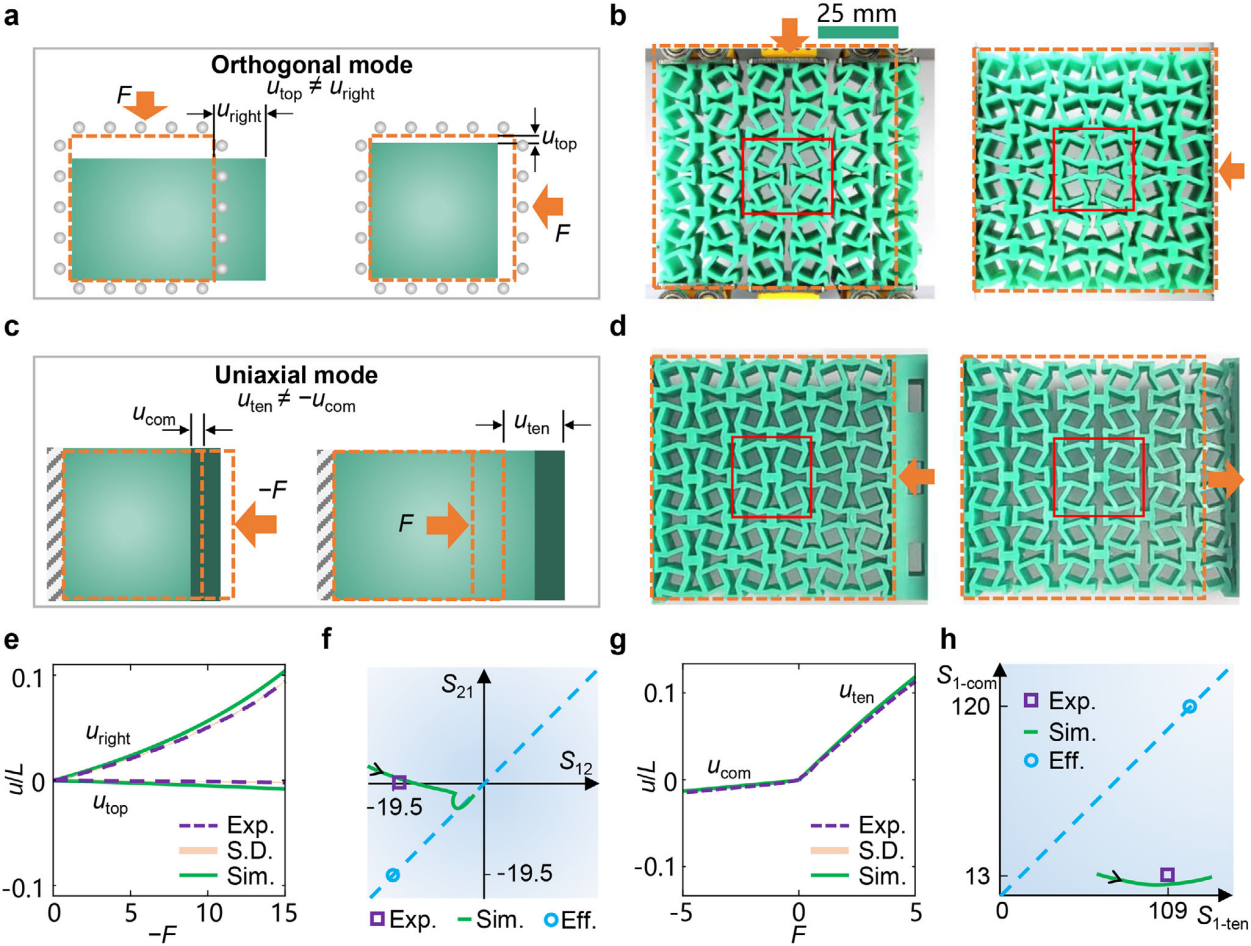
Orthogonal and uniaxial nonreciprocal modes. a) Schematic illustration of the orthogonal nonreciprocal mode. b) Deformation patterns of the metamaterial under forces of −15 and 15 N on the top and right, respectively. The undeformed structure is represented by orange dashed lines, and one cell is boxed by red lines. c) Schematic illustration of the uniaxial nonreciprocal mode. d) Deformation patterns of the metamaterial under transverse forces of −5 N and 5 N on the right, respectively. e) Orthogonal nonreciprocal responses. The solid and dashed lines are the data from simulations (Sim.) and means of experiments (Exp.), respectively. The structure and constraint conditions in simulations are the same as those in experiments. The shadow under the dashed lines is the sample standard deviation of experiments (S.D.) based on five repeated tests. f) *S*_12_ and *S*_21_. For the square, *S*_12_ and *S*_21_ are tested by experiments under transverse and longitudinal compressions, respectively. For the green line, *S*_12_ and *S*_21_ are evaluated by cells with different *θ* from −*π*/6 to *π*/6. The black arrow on the green line represents the trend of the change in *θ*. For the circle, *S*_12_ and *S*_21_ are effective values (Eff.) calculated by the method of representative volume element. The blue dashed line represents the reciprocal response, and its slope is 1. g) Uniaxial nonreciprocal responses. h) *S*_1‐ten_ and *S*_1‐com_, which are *S*_1_ evaluated by tension and comprssion, respectively.

**Figure 3:**
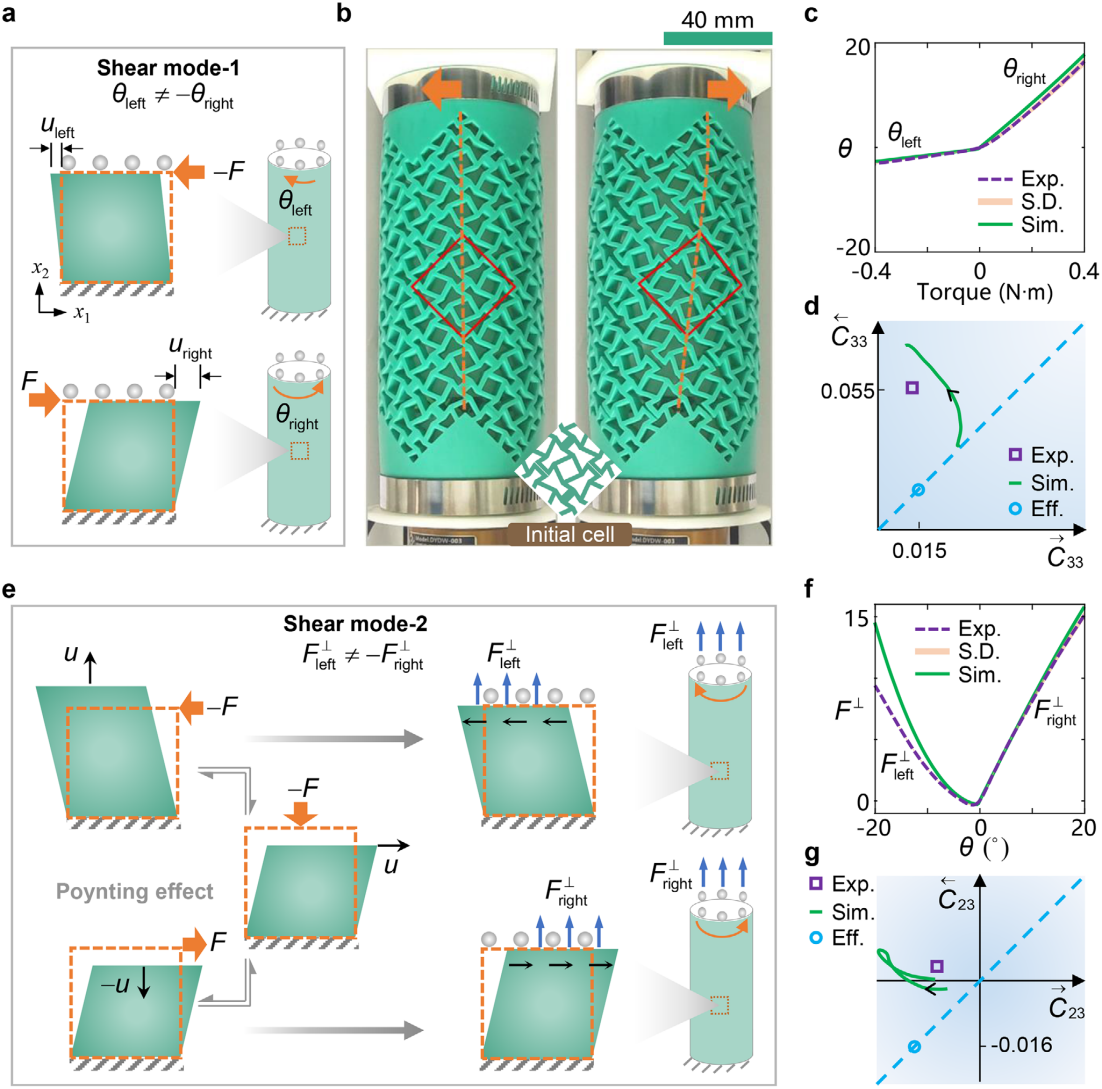
Shear nonreciprocal modes. a) Schematic illustration of the nonreciprocal mode on the shear strain. *u*_left_ and *u*_right_ are shear displacements to the left and right under the same forces, respectively. θ_left_ and θ_right_ are rotation angles to the left and right under the same torques, respectively. b) Torsion deformation patterns of the metamaterial tube under −0.4 and 0.4 N∙m, respectively. The undeformed cell in the plane is inserted in the bottom. c) θ_left_ and θ_right_ evaluated by experiments and simulations. The sample standard deviations of the experiments are obtained from five repeated tests. d) Stiffness tensor *C*_33_. For the square, ${\overset{\leftarrow }{C}}_{33}$ and ${\vec{C}}_{33}$ are *C*_33_ tested under rotation to the left and right, respectively. e) Schematic illustration of the nonreciprocal mode on the Poynting effect. $F_{\mathrm{left}}^{⊥}$ and $F_{\mathrm{right}}^{⊥}$ are reaction forces of the metamaterial tube to the top constraint when the tube rotates left and right, respectively. f) $F_{\mathrm{left}}^{⊥}$ and $F_{\mathrm{right}}^{⊥}$ evaluated by experiments and simulations. g) Stiffness tensor *C*_23_. ${\overset{\leftarrow }{C}}_{23}$ and ${\vec{C}}_{23}$ are *C*_23_ tested under rotation to the left and right, respectively.

**Figure 4:**
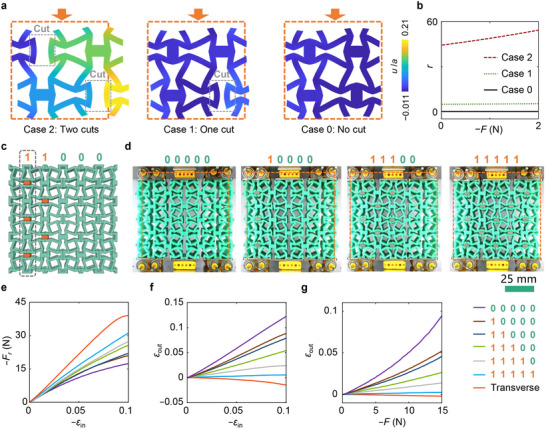
Reprogrammable nonreciprocity via reconfigurable cuts. a) Deformation patterns of the unit cells with different numbers of predefined cuts under longitudinal compression strain of 10%. b) Constitutive asymmetry. c) Encoding strategy using external constraints. d) deformations of the metamaterial under different encodings. The compressive strain is 10%. The undeformed structure is represented by the dashed line. e) Reprogrammable reaction force by encoding different sets of binary instructions onto the metamaterial. *F* is the compressive force. ε_in_ = *u*_in_/*L*. *u*_in_ is the input displacement in the compressive direction. *L* is the side length of the square sample. f) Reprogrammable transmission of the displacement field. ε_out_ = *u*_out_/*L*. *u*_out_ is the output displacement in the direction that is perpendicular to the compressive force. g) Reprogrammable static nonreciprocity. In experiments, the binary instructions are listed in the legends with different markers, all of which are built for longitudinal compression. The responses of experiments under transverse compression are described as 'Transverse' in the legends. The lines are the means of experiments with five repeated tests. The sample standard deviations are provided in Figure S8 (Supporting Information).

In Figure 1e1,e2, we present the global elastic moduli and Poisson's ratios under small strains of 0.1% in terms of proper ranges of the beam length *b* and rotation angle *θ* of the square. In 0.2*a* < *b* < 0.3*a* and −*π*/6 < *θ* < *π*/6, the longer beam for specified *θ* yields the larger modulus *E*_1_ and *E*_2_. The values of Poisson's ratios can realize an unusual combination, including *v*_12_ > 1 and *v*_21_ ≤ 0, which is not supported by the constitutive symmetry *v*_12_/*E*_2_ = *v*_21_/*E*_1_ with positive elastic moduli.^[^ ^38^ ^]^ The metamaterial can realize unidirectional and amplified transmission of displacement fields via this combination of Poisson's ratios. Unless otherwise stated, we use the unit cells with *w*_1_ = 0.06*a*, *w*_2_ = 0.2*a*, *w*_3_ = 0.0002*a*, *b* = 0.28*a*, and *θ* = *π*/9 for the analysis of different nonreciprocal modes (**Figures** **2**–**4**).

### 3.2 Orthogonal Nonreciprocal Mode

Before evaluating the effects of cuts, we confirm that including hyperelastic nonlinearity of the substrate material has negligible influence on the nonreciprocal behavior (Note S6, Supporting Information), and geometrical nonlinearity dominates the nonreciprocity before contact. The schematic illustration of the orthogonal nonreciprocal mode associated with *S*_12_ and *S*_21_ is presented in **Figure** **2**a. The nonreciprocal response $u_{\mathrm{top}}\neq u_{\mathrm{right}}$ is obtained under force *F* in two orthogonal directions, respectively. As *S*_12_ = −*v*_12_/(*E*_2_/*E*_0_) and *S*_21_ = −*v*_21_/(*E*_1_/*E*_0_), based on Equation (1), we obtained the constitutive asymmetry indicator *r* of the typical case *w*_3_ = 0.02*a*. As shown in Figure 1d, contact at the cuts makes *r* increase sharply, and the transition point depends on the width of the cut clearance. In addition, significant nonreciprocity with *r* > 30 can be achieved under small strain. Furthermore, we evaluate *r* under 0.2*a* < *b* < 0.3*a* and −*π*/6 < *θ* < *π*/6, as shown in Figure 1e3. It shows that the nonreciprocity is more sensitive to *θ*, and the nonreciprocity at *θ* = *π*/6 is larger than that at *θ* = −*π*/6.

We fabricated a sample comprising 3×3 cells to demonstrate the orthogonal nonreciprocal mode. As shown by the circle point in Figure 2f, if no nonlinearity is considered, we have *S*_12_ = *S*_21_ = −19.5 by the method of representative volume element (RVE, Note S5, Supporting Information),^[^ ^50^ ^]^ obeying the reciprocity and constitutive symmetry. As shown in Figure 2b, when applying a compression force *F* = 15 N on the top surface, we obtain the displacement *u*_right_ = 7.1 mm on the right surface. In contrast, when applying the same compression force on the right surface, the displacement on the top surface is *u*_top_ = 0.15 mm. In experiments, the two sides for applying force have two sliding boundary conditions identical to simulations, realized via using bearings. The other pair of sides is free to simplify the experiments. Based on a small input strain of 1%, the experiments give *S*_12_ = 18.6 and *S*_21_ = 0.29 (the square in Figure 2f). Therefore, the point (*S*_12_, *S*_21_) is no longer lying on the straight line for *S*_12_ = *S*_21_: Strong orthogonal nonreciprocal deformation is obtained.

### 3.3 Uniaxial Nonreciprocal Mode

The design can also offer a uniaxial nonreciprocal mode that leads to different displacements *u*_com_ and *u*_ten_ under an equal compressive and tensile force *F*, as presented in Figure 2c. It breaks the reciprocity − *Fu*_com_ = *Fu*_ten_ Such a uniaxial nonreciprocal mode can be characterized by the generalized compliance tensor *S*_1_ = 1/(*E*_1_/*E*_0_) (Note S1, Supporting Information).

A sample comprising 3×3 cells is fabricated to demonstrate the unidirectional nonreciprocal mode. On the left and right sides of the sample, there are two layers of auxiliary material utilized for tension experiments. In a reciprocal system, whether the structure is compressed or stretched, *S*_1_ is invariant as shown by the circle points in Figure 2h, where in both compression and tension *S*_1‐com_ = *S*_1‐ten_ = 120. As shown in Figure 2d, we apply a compressive and tensile force of 5 N on the right surface, respectively. The displacements in compression and tension are *u*_com_ = −1.1 mm and *u*_ten_ = 8.5 mm, respectively. This gives *S*_1‐com_ = 13 and *S*_1‐ten_ = 109 (the square in Figure 2h), breaking the invariance of the constitutive tensor *S*_1_. Therefore, the uniaxial nonreciprocal deformation is obtained, though the geometrical mirror symmetry is not broken (Figure S2, Supporting Information). The tensile stiffness is much smaller because contact is not activated at cuts under tension.

### 3.4 Shear Nonreciprocal Mode on Shear Displacement

The nonreciprocal shear mode is presented in **Figure** **3**a, denoted by $-Fu_{\mathrm{left}}\neq Fu_{\mathrm{right}}$, in which *u*_left_ (or *u*_right_) is the shear displacement on the left (or right) under the left (or right) shear force *F*. The shear behavior in the plane is mapped into a tube, and then the nonreciprocal shear responses are transformed into the rotation angles θ_left_ and θ_right_ under an equal torque. The shear nonreciprocal mode can be described by the dimensionless stiffness tensor *C*_33_ = (σ_*xy*_/γ_*xy*_)/*E*_0_, where σ_*xy*_ is shear stress and γ_*xy*_ is shear strain, respectively (Notes S1 and S7, Supporting Information).

We obtain a shear nonreciprocal mode by rotating the cell by 45°, as shown in Figure 3b. The metamaterial tube includes 5×3 unit cells, where cuts break the mirror symmetry between two periodic supercells corresponding to shear strains applied in two opposite directions, respectively (Figure S3, Supporting Information). In a reciprocal system, whether the structure shears to the left or right, *C*_33_ is invariant as shown by the circle points in Figure 3d, where in both two directions ${\overset{\leftarrow }{C}}_{33}={\vec{C}}_{33}=0.015$. When applying the same torque 0.4 N∙m in two opposite directions, the torsion angles are θ_left_ = −3.1° and θ_right_ = 16.5°, respectively. The experiments give ${\overset{\leftarrow }{C}}_{33}=0.055$ and ${\vec{C}}_{33}=0.013$, breaking the invariance of the constitutive tensor *C*_33_. The ${\overset{\leftarrow }{C}}_{33}$ is much larger because contact at the cuts enhances the resistance of the structure to turning left.

### 3.5 Shear Nonreciprocal Mode on the Poynting Effect

Furthermore, we show a shear nonreciprocal mode on the Poynting effect,^[^ ^51^ ^]^ in Figure 3e. When a tube with an anisotropic material is twisted in two opposite directions about its axis, respectively, the Poynting effect causes the tube to expand and shrink axially, respectively. When the upper and lower ends of the tube are restricted from axial displacement, the Poynting effect makes the tube yield a reaction force (compressive or tensile force) to the top constraint, i.e., $F_{\mathrm{left}}^{⊥}$ and $F_{\mathrm{right}}^{⊥}$ as shown in Figure 3e. In a reciprocal system with anisotropic material, the $F_{\mathrm{left}}^{⊥}$ and $F_{\mathrm{right}}^{⊥}$ are the combination of compressive and tensile forces. However, the shear nonreciprocal mode on the Poynting effect makes $-F_{\mathrm{left}}^{⊥}\neq F_{\mathrm{right}}^{⊥}$, enabling both $F_{\mathrm{left}}^{⊥}$ and $F_{\mathrm{right}}^{⊥}$ are compressive forces as shown in Figure 3e. The Poynting effect on $F_{\mathrm{left}}^{⊥}$ and $F_{\mathrm{right}}^{⊥}$ can be characterized by the dimensionless stiffness tensor *C*_23_ = σ_*yy*_/γ_*xy*_, where σ_*yy*_ is the *y*‐axis stress under pure shear strain [0, 0, γ_*xy*_]^*T*^ (Notes S1 and S7, Supporting Information).

In a reciprocal system, *C*_23_ is invariant as shown by the circle points in Figure 3g, where in both two shear directions ${\overset{\leftarrow }{C}}_{23}={\vec{C}}_{23}=-0.016$. When applying the torsion angle 20° in two opposite directions, the reaction forces to the top constraint are $F_{\mathrm{left}}^{⊥}$= 9.4 N∙m and $F_{\mathrm{right}}^{⊥}$= 15.6 N∙m, respectively, both of which are compressive forces. The experiments give ${\overset{\leftarrow }{C}}_{23}$ = 0.0035 and ${\vec{C}}_{23}$ = −0.01, ${\vec{C}}_{33}=0.013$, breaking the invariance of the constitutive tensor *C*_23_. When the tube twists to the right, the clearance at the cuts increases, and the tube tends to expand longitudinally. However, when the tube twists to the left, contact at the cuts resists longitudinal contraction, causing the tube to generate a tendency to expand longitudinally once again.

### 3.6 Reprogrammable Nonreciprocity via Reconfigurable Cuts

The analyses above demonstrate that our metamaterial can present different nonreciprocal modes for the same unit cell. However, the nonreciprocal properties remain when the metamaterial is fabricated. Programmability can expand the capability for reaching broader material parameters. Though one can reconfigure the properties via using shape‐memory materials, a more convenient way for realizing tunability in our metamaterial is encoding the cuts, i.e., setting different numbers of predefined cuts, simply by using external constraints like clamps.

Every unit cell can present three states: two cuts are predefined (Case 2); one cut is retained and the other is constrained (Case 1); and no cut is retained (Case 0). Properties present in Case 2 have been analyzed above. In Case 1, the compressive modulus *E*_2_ is larger and *v*_12_ is smaller than those in Case 2, reducing the difference between *S*_12_ and *S*_21_. Under small strain, the constitutive asymmetry *r* = 4.9 in Case 1 is smaller than 44.4 in Case 2, manifesting weak nonreciprocity. When there is no predefined cut in the cell (case 0), *C*_4_ rotation symmetry emerges, and no contact nonlinearity happens, resulting in a symmetrical constitutive relation, as shown in Figure 4b.

In the cellular array, every cut has two states: constrained or retained. Therefore, there are 2*^n^* states in theory, where *n* denotes the total number of cuts. Here, we define the state of a column of cuts to succinctly show the programmability. If a column of cuts is all constrained, the state of this column is “1”. Inversely, its state is “0”. We considered six cases in the cellular array with 5 columns of cuts. By encoding different sets of binary instructions, the metamaterial requires different levels of compressive forces *F* to reach the same input displacements *u*_in_ as shown in Figure 4e, which demonstrates the reprogrammable strain‐stress response. The force‐displacement response of each set of binary instructions is reversible until the system is reprogrammed. Similar to the reaction force, the transmission of the displacement fields is reprogrammable, as shown in Figure 4f. When coded with the set of all zero instructions, the longitudinal input displacement can be amplified. The metamaterial possesses the largest static nonreciprocity when all cuts are retained (case 00000), as shown in Figure 4g. The static nonreciprocity can be reduced by restricting more predefined cuts, and the static nonreciprocity is reprogrammable via different sets of binary instructions.

## 4 Potential Applications of Cut‐Enabled Nonreciprocal Metamaterials

The nonreciprocal modes offer the designed metamaterials to have direction‐dependent stiffness, one‐way transmission of displacement fields, and unique shear responses. Meanwhile, the reprogrammability allows for the tuning of these properties. The designed metamaterials hold promise for applications in auxiliary exoskeletons outside the soft robot bodies (Note S9, Supporting Information), mechanical integrated circuits, energy harvesters, and motion limiters.

**Figure 5:**
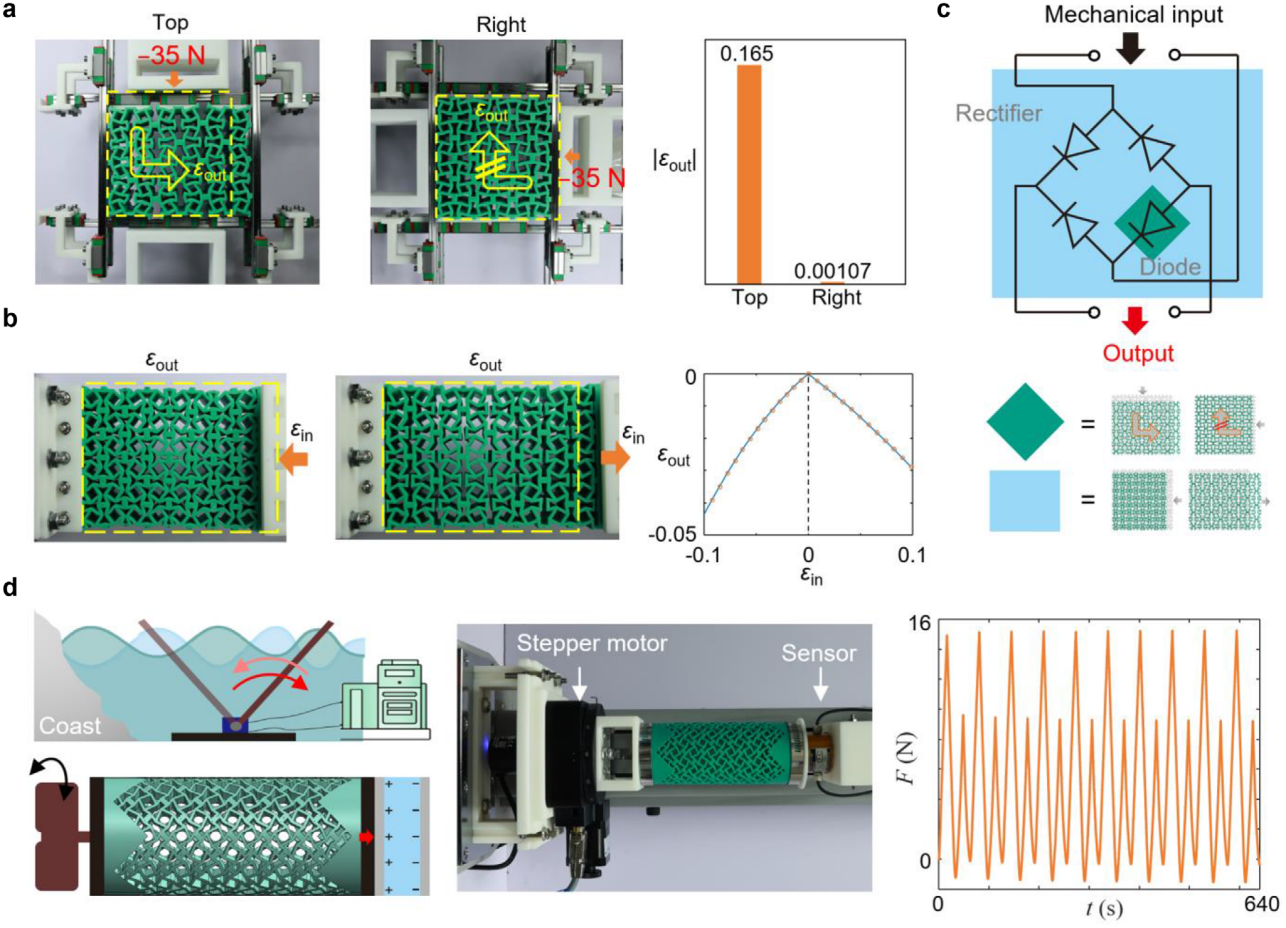
Potential applications of designed nonreciprocal metamaterials. a) Transmissions of displacement fields in experiments. The dashed lines represent the undeformed structures. The parameters of the microstructure are *w*_1_ = 0.06*a*, *w*_2_ = 0.2*a*, *b* = 0.25*a*, and *θ* = *π*/12. *w*_3_ = 0.0002*a*. The thickness of the sample is 40 mm. b) Longitudinal strains under transverse compression and tension from experiments, respectively. The parameters of the microstructure are *w*_1_ = 0.06*a*, *w*_2_ = 0.2*a*, *b* = 0.27*a*, and *θ* = *π*/6. *w*_3_ = 0.0002*a*. The thickness of the sample is 30 mm. c) The analogies between metamaterials and electronic components for the mechanical diode and rectifier. d) Wave energy harvester and mechanical experiments. The metamaterial tube is the same as that in Figure 3b. The forces on the sensor include 10 cycles of rotation in 640 s. The maximum torsion angle is 20°.

Mechanical integrated circuits with mechanical logic elements can advance the information processing of autonomous engineered matter.^[^ ^52^, ^53^, ^54^ ^]^ The designed nonreciprocal metamaterial in **Figure** **5**a experimentally shows one‐way transmissions of displacement fields, functioning as a mechanical diode. The experimental framework that provides sliding boundary conditions is built based on guide rails and sliders (Figure S9, Supporting Information). In the two loading cases of Figure 5a, the same boundary conditions are used. Another designed metamaterial in Figure 5b contracts longitudinally when stretched or compressed transversely, acting as a mechanical rectifier. Thus, these designed metamaterials have special mechanical responses for mechanical diodes and mechanical rectifiers that serve as functional modules to improve the scalability of mechanical integrated circuits.

Wave energy harvesting has garnered significant attention. We can utilize the waves near the coast to drive the rotation of metamaterials as shown in Figure 5d, and then piezoelectric materials transform the axial forces (Figure 3e) into electrical energy.^[^ ^29^ ^]^ In the experiments, we use a stepper motor to simulate the driving forces of waves, and a force sensor is used to show the axial forces. For general anisotropic materials, the reciprocal Poynting effect ($-F_{\mathrm{left}}^{⊥}=F_{\mathrm{right}}^{⊥}$) makes the structure generate tension and pressure on the sensor when rotating clockwise and counterclockwise, respectively. The nonreciprocal Poynting effect in this study makes the metamaterial exert pressure on the piezoelectric material at one end, (almost) regardless of whether the structure rotates clockwise or counterclockwise, as shown in Figure 5d, realizing the conversion of wave energy into electrical energy. The minor tensile force in each cycle is due to the gap of the cuts. Furthermore, the varying shear moduli under clockwise and counterclockwise rotation, as shown in Figure 3d, allow the metamaterial to function as a rotation limiter for one‐way door opening.

## 5 Conclusion

In summary, we have demonstrated how multimodal and reprogrammable static nonreciprocity can be realized by cellular metamaterials with reconfigurable cuts. In each cell, the cut exhibits “contact” and “noncontact” states under different loads to realize strong static nonreciprocity. The metamaterial integrates four nonreciprocal modes, including the orthogonal, uniaxial, and two shear modes. We found that the multimodal nonreciprocity can be characterized by four pairs of constitutive tensors: *S*_12_ and *S*_21_, *S*_22_ in tension and compression, *C*_33_ in left‐ and right‐hand patterns, and *C*_23_ in left‐ and right‐hand patterns, which provide a framework for studying static nonreciprocal phenomena. The constitutive tensors that characterize the multimodal nonreciprocity provide great guidance for the inverse design methods, such as topology optimization, which have shown powerful abilities to design constitutive tensors.^[^ ^32^ ^]^ Moreover, we introduce external constraints to reconfigure the layout of cuts, and build a reprogrammable strategy to manipulate the stiffness, unidirectional displacement transmission, and nonreciprocity.

Compared to the existing static nonreciprocal structures,^[^ ^28^ ^]^ with a uniaxial mode, the cut‐enabled metamaterials present multimodal and reprogrammable static nonreciprocity by introducing contact nonlinearity, enriching the static nonreciprocal responses. The constitutive tensors for multimodal nonreciprocity describe the structural bulk behaviors, which are different from the topologically induced edge modes with zero energy.^[^ ^28^ ^]^ The cut‐enabled metamaterials are topologically trivial due to the absence of topologically induced edge modes.

The polarized stiffness, one‐way transmission of displacement fields, unusual Poynting effect, and reprogrammbility shown by the cut‐enabled metamaterials broaden the potential applications of static nonreciprocity in soft robotics, mechanical logic, and energy harvesters.

## 6 Experimental Section

### Sample Fabrication

All samples were manufactured by molding. First, a light‐curing 3D printer (ZRapid) was applied to manufacture the prototypes of the designed cylindrical shells. Then, the prototypes were put into a silica gel solution, and vacuum equipment was used to eliminate the air in the solution. Next, the molds were obtained after removing the prototypes from the cured silica gel solution. Finally, the molds were placed into a vacuum pouring machine that was used to inject mixed polyurethane raw material into the molds. When the material was cured, the molds were removed from the samples. In Figures 2, 3, 4, the parameters of the unit cells were *w*_1_ = 0.06*a*, *w*_2_ = 0.2*a*, *w*_3_ = 0.0002*a*, *b* = 0.28*a*, and *θ* = *π*/9. The samples in Figure 2 and Figure 4 include 3×3 unit cells with *a* = 25 mm. The thicknesses of the samples in Figure 2 and Figure 4 were 25 mm. The metamaterial tube includes 5×3 unit cells with *a* = 25 mm. The thickness of the metamaterial tube was 5 mm.

### Experimental Measurement

The compression tests were performed using a single‐column tabletop testing system, IMADA ZTA with a 500 N loading cell. In torsion tests, the torque and compression forces were obtained in a multidimensional sensor, DAYSENSOR DYDW‐003, of which load ranges were 3 N·m and 50 N, respectively. The rotary table was YIXUAN YXRA100, of which the maximum static torque was 25 N·m. The angle sensor was OIDENCODER OID‐3806D, of which the angle range was 360°. The tests were monitored using a camera (Canon EOS R7) with 3840×2160 pixels. The videos were analyzed by the motion analysis software, Tracker. More detailed information on the experiments and the calculation of constitutive tensors based on experimental data is provided in Note S7 (Supporting Information). The detailed boundary conditions of metamaterials and the analysis of the influence of their changes are provided in Note S8 (Supporting Information).

### Numerical Simulation

The simulations of the samples of the metamaterials were performed with FEM (Finite Element Method) in ABAQUS 2023, where the structures and boundary conditions were the same as those in the experiments. In the simulations of unit cells with periodic boundary conditions, different strains were applied to the unit cell to simulate the deformations of different nonreciprocity modes as shown in Figure S4 (Supporting Information). A Mooney‐Rivlin model was used for the material nonlinearity of hyperelasticity, of which parameters *c*_10_ and *c*_01_ were 3.4E5 and 4.9E4 Pa, respectively. In the simulations of metamaterials, the elastic moduli in Figure 2b,d, and Figure 3b were 2.6, 2.3, and 20.7 MPa, respectively, and the Poisson's ratios were 0.48. The simulations of unit cells of the metamaterials and the RVE were performed with FEM in COMSOL Multiphysics 6.2. Quadrilateral elements were used in simulations of 2D structures with the assumption of plane stress. Hexahedral elements were applied to the simulations of the metamaterial tube.

### Statistical Analysis

Displacement and constitutive tensors from experiments were transformed into dimensionless values based on the sizes of undeformed structures and the elastic moduli of the substrate materials, respectively. The means and sample standard deviations of the experiments were obtained from five repeated tests. MATLAB was used to calculate the mean and sample standard deviation of experiments.

## Conflict of Interest

The authors declare no conflict of interest.

## Supporting information



Supporting Information

## Data Availability

The data that support the findings of this study are available from the corresponding author upon reasonable request.
