# Supplementary material for: Cut‐Enabled Mechanical Metamaterials for Multimodal and Reprogrammable Static Nonreciprocity
Source: Adv Sci (Weinh). 2025 Aug 20;12(42):e03455. doi: 10.1002/advs.202503455 (PMC12622417; doi:10.1002/advs.202503455)
Supplement: Supplementary file 1 — Supporting Information [file ADVS-12-e03455-s001.docx]

**Supplementary Information**

**Cut-Enabled Mechanical Metamaterials for Multimodal and Reprogrammable Static Nonreciprocity**

Jinhao Zhang^1, 5, *^, Shuo Zhang^2, 5^, Xiao Zhou^1, 5^, Yueqi He^1^, Fengwen Wang^3^, Dianlong Yu^1^,

Yu Jiang^1, *^, Mi Xiao^4, *^, Xin Fang^1, *^

^1^National Key Laboratory of Equipment State Sensing and Smart Support, College of Intelligence Science and Technology, National University of Defense Technology, 410073 Changsha, China

^2^State Key Laboratory of Bioinspired Interfacial Materials Science, Bioinspired Science Innovation Center, Hangzhou International Innovation Institute of Beihang University, Hangzhou, 311115 China

^3^Department of Civil and Mechanical Engineering, Technical University of Denmark, Koppels Allé, Building 404, 2800 Kongens Lyngby, Denmark

^4^State Key Laboratory of Intelligent Manufacturing Equipment and Technology, Huazhong University of Science and Technology, 430074 Wuhan, China

^5^These authors contributed equally: Jinhao Zhang, Shuo Zhang, Xiao Zhou

^*^Corresponding authors. Email: [jinhaomail@nudt.edu.cn](mailto:jinhaomail@nudt.edu.cn), jiangyu@nudt.edu.cn, xiaomi@hust.edu.cn, [xinfangdr@sina.com](mailto:xinfangdr@sina.com)

**Supplementary Note 1**

**Deformation modes of different constitutive tensors**

In 2D linear elastic mechanics, the relation between strain and stress is $\mathbf{\varepsilon}=\mathbf{S\sigma}$:

$$\left[\begin{matrix} \varepsilon_{xx} \\ \varepsilon_{yy} \\ \gamma_{xy} \end{matrix}\right]=\left[\begin{matrix} S_{11} & S_{12} & S_{13} \\ S_{21} & S_{22} & S_{23} \\ S_{31} & S_{32} & S_{33} \end{matrix}\right]\left[\begin{matrix} \sigma_{xx} \\ \sigma_{yy} \\ \sigma_{xy} \end{matrix}\right]$$

The constitutive tensors are associated with three deformation behaviors, including orthogonal, uniaxial, and shear modes. In uniaxial mode, $\sigma_{xx}\neq0$, $\sigma_{yy}=0$ and $\sigma_{xy}=0$, or $\sigma_{xx}=0$, $\sigma_{yy}\neq0$ and $\sigma_{xy}=0$. Then the uniaxial strain is evaluated by $\varepsilon_{xx}=S_{11}\sigma_{xx}$ or $\varepsilon_{yy}=S_{22}\sigma_{yy}$. In orthotropic mode, $\sigma_{xx}\neq0$, $\sigma_{yy}=0$ and $\sigma_{xy}=0$, or $\sigma_{xx}=0$, $\sigma_{yy}\neq0$ and $\sigma_{xy}=0$. Then the orthotropic strain is evaluated by $\varepsilon_{yy}=S_{21}\sigma_{xx}$ and $\varepsilon_{xx}=S_{12}\sigma_{yy}$. In shear mode, $\sigma_{xx}=0$, $\sigma_{yy}=0$ and $\sigma_{xy}\neq0$, the shear strain is calculated by $\gamma_{xy}=S_{33}\sigma_{xy}$, and the Poynting effect is evaluated by $\varepsilon_{yy}=S_{23}\sigma_{xy}$ or $\varepsilon_{xx}=S_{13}\sigma_{xy}$. To reduce the difficulty of the experiments, the shear mode can be transformed based on $\mathbf{\sigma}=\mathbf{C\varepsilon}$, where $\mathbf{C}=\mathbf{S}^{-1}$ and **C** includes *C_ij_* (*i*=1, 2, 3 and *j*=1, 2, 3). Then, the shear mode can be tested via $\varepsilon_{xx}=0$, $\varepsilon_{yy}=0$ and $\gamma_{xy}\neq0$. Then the shear stress is calculated by $\sigma_{xy}=C_{33}\gamma_{xy}$, and the Poynting effect is evaluated by $\sigma_{yy}=C_{23}\gamma_{xy}$ or $\sigma_{xx}=C_{13}\gamma_{xy}$.

**Supplementary Note 2**

**Nonreciprocity with constitutive asymmetry**

In the orthotropic linear elasticity, the compliance tensors are

$$\left[\begin{matrix} S_{11} & S_{12} & S_{13} \\ S_{21} & S_{22} & S_{23} \\ S_{31} & S_{32} & S_{33} \end{matrix}\right]=\left[\begin{matrix} 1/E_{1} & -v_{12}/E_{2} & 0 \\ -v_{21}/E_{1} & 1/E_{2} & 0 \\ 0 & 0 & 1/G \end{matrix}\right]$$

where *G* is the shear modulus. The constitutive symmetry ($S_{12}=S_{21}$) determines

$$v_{12}/E_{2}=v_{21}/E_{1}$$

The elastic moduli can be evaluated via stress and strain, i.e., $E_{1}=(F_{1}/A)/(u_{1}/a)$ and $E_{2}=(F_{2}/A)/(u_{2}/a)$. $F_{1}$ (or $F_{2}$) is the reaction forces under the strain $u_{1}/a$ (or $u_{2}/a$) in the *x* (or *y*)-axis. *A* is the sectional area perpendicular to the reaction forces. For the square unit cell, Poisson’s ratio $v_{12}$ (or $v_{21}$) is the negative value of the ratio between the output displacement $u_{2\to1}$ (or $u_{1\to2}$) in the *x* (or *y*)-axis and input displacement $u_{2}$ (or $u_{1}$) in the *y* (or *x*)-axis. Then Eq. can be rewritten as

$$\frac{-u_{2\to1}}{u_{2}}/\frac{F_{2}a}{Au_{2}}=\frac{-u_{1\to2}}{u_{1}}\text{/}\frac{F_{1}a}{Au_{1}}$$

which can be simplified as a reciprocal formulation:

$$F_{1}u_{2\to1}=F_{2}u_{1\to2}$$

Thus, if the constitutive symmetry is broken ($S_{12}\neq S_{21}$) in the structure, static nonreciprocity emerges, i.e., $F_{1}u_{2\to1}\neq F_{2}u_{1\to2}$.

**Supplementary Note 3**

**Symmetry of the designed metamaterials**

We analyze the general symmetry of the designed metamaterials. The topology of the designed metamaterials has *C*_4_ rotation symmetry and mirror symmetry (Supplementary Fig. 1). When cuts are introduced, the *C*_4_ rotation symmetry of the designed metamaterials is broken, but the mirror symmetry is retained (Supplementary Fig. 2). When we rotate the metamaterials by 45°, the cuts break the mirror symmetry between two periodic supercells for left and right shear strains (Supplementary Fig. 3).

**
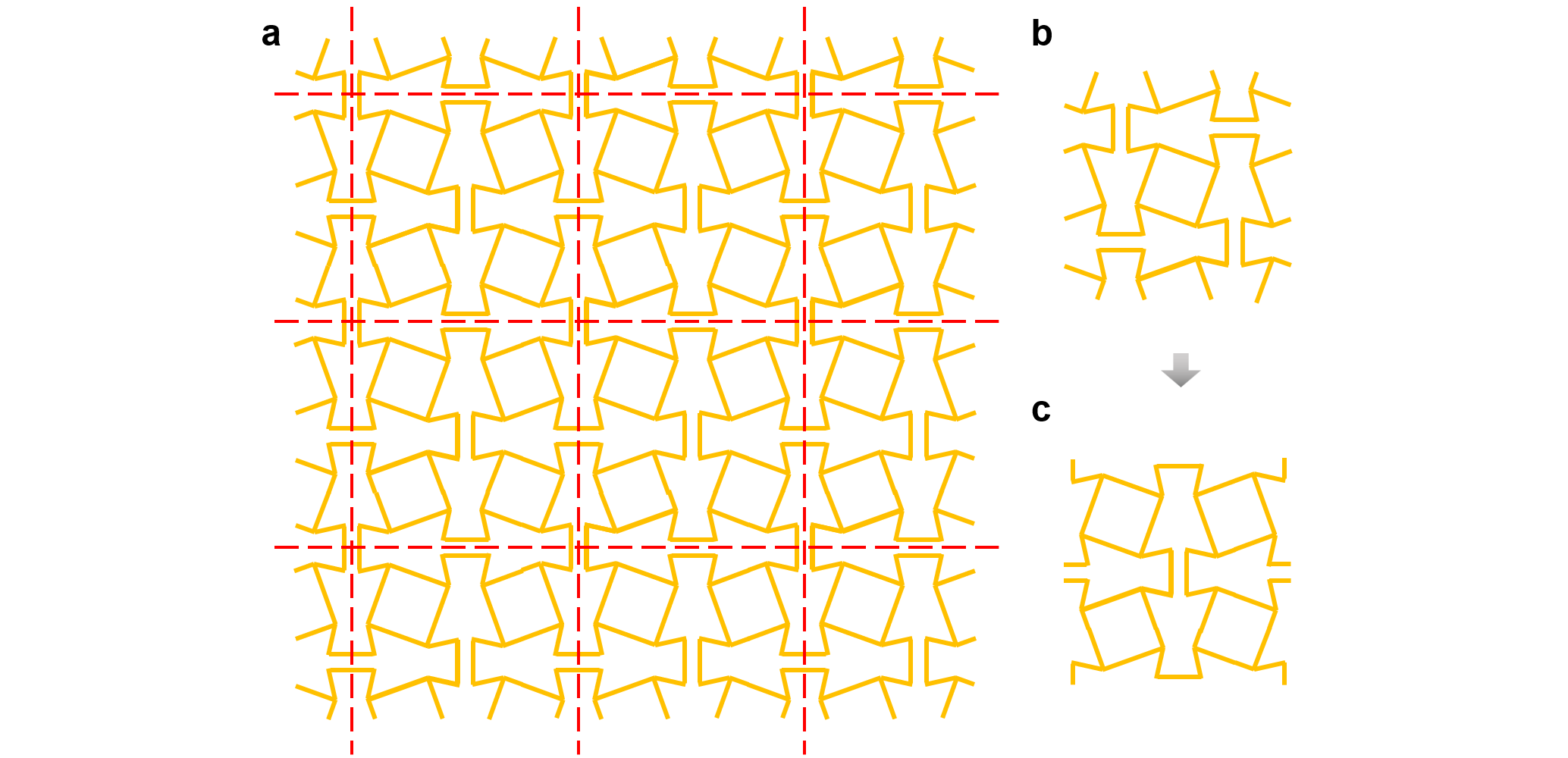
**

**Supplementary Fig. 1: The topology of the design metamaterials. a**, Topology of 3×3 unit cells. The red lines are the boundaries of the unit cells. **b**, *C*_4_ rotation symmetry of topology. **c**, Anti-chirality of the topology.

**
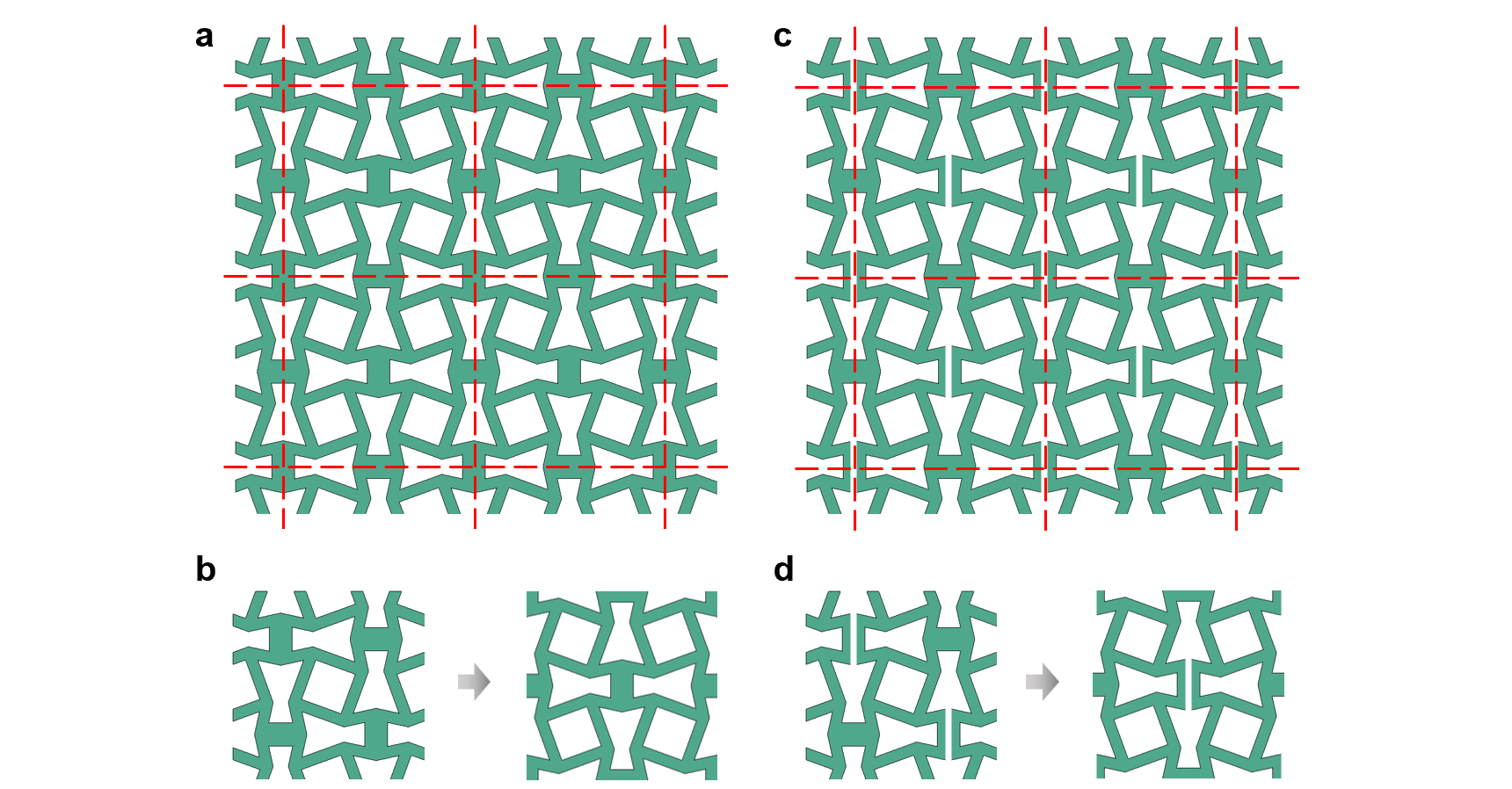
**

**Supplementary Fig. 2: Mirror symmetries of the designed metamaterials. a**, 3×3 unit cells without cuts. The red lines are the boundaries of the unit cells. **b**, *C*_4_ rotation symmetry and mirror symmetry of the metamaterials without cuts. **c**, 3×3 unit cells with cuts. **d**, *C*_2_ rotation symmetry and mirror symmetry of the metamaterials with cuts.


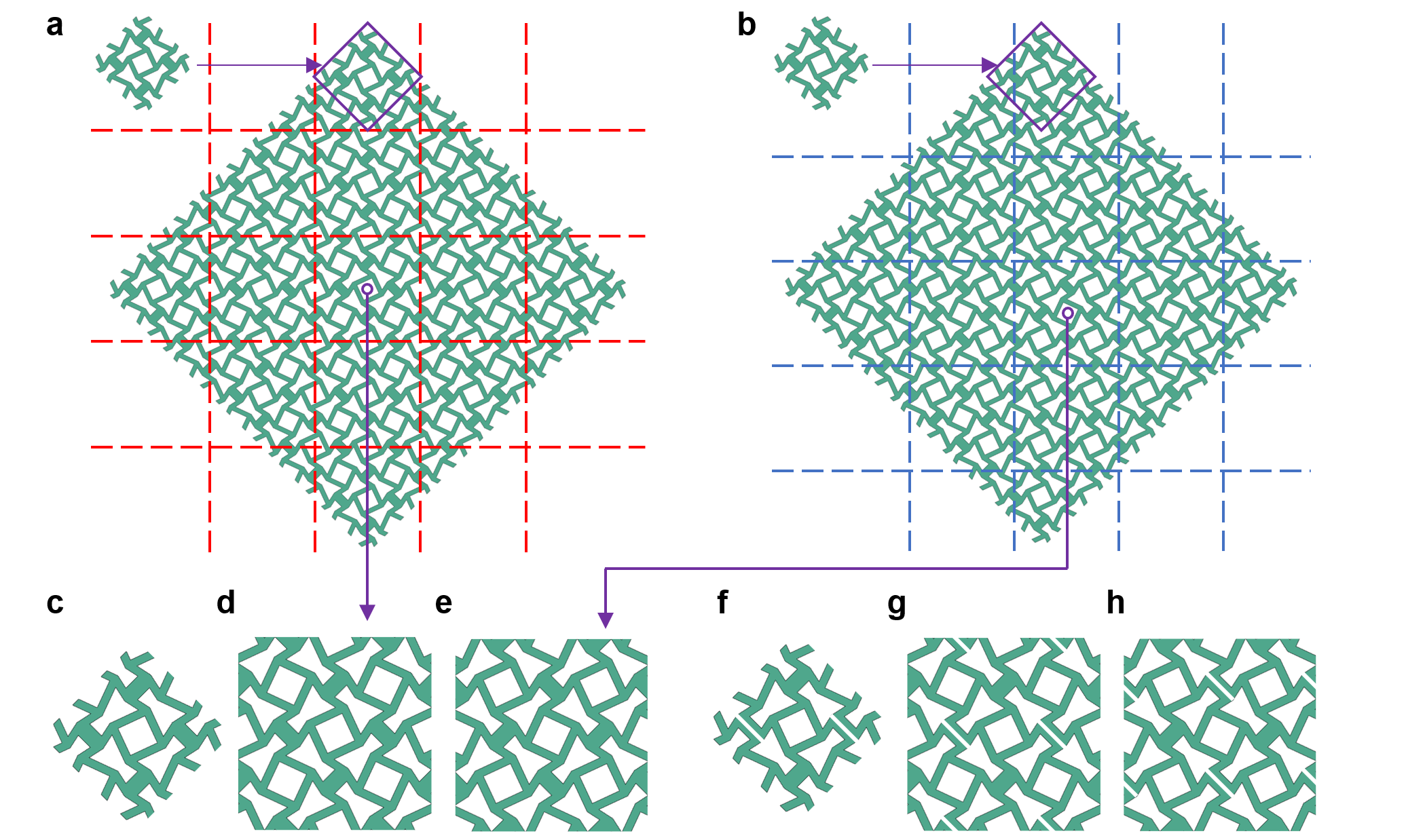


**Supplementary Fig. 3: Mirror symmetries of 5×5 unit cells that are rotated** **45 degrees. a**, **b**, Different periodic cells. **c**, Unit cell without cut that are rotated $45^{\circ}$. **d**, **e**, Mirror symmetry between two different periodic supercells. **f**, Unit cell with cuts that are rotated $45^{\circ}$. **g**, **h**, Broken mirror symmetry between two different periodic supercells with cuts.

**Supplementary Note 4**

**Periodic boundary conditions**

For the metamaterials with periodically arranged unit cells, Poisson’s ratio *v*_12_ and elastic modulus *E*_2_ in the orthotropy are evaluated based on the periodic boundary conditions in Eq. as shown in Supplementary Fig. 4a, and Poisson’s ratio *v*_21_ and elastic modulus *E*_1_ in the orthotropy are evaluated based on Eq. as shown in Supplementary Fig. 4b. Based on $S_{11}=1/E_{1}$, $S_{22}=1/E_{2}$, $S_{12}=-v_{12}/E_{2}$ and $S_{21}=-v_{21}/E_{1}$, we can obtain *S*_11_, *S*_22_, *S*_12_ and *S*_12_. For *C*_33_ and *C*_23_, we use the periodic boundary conditions in Eq. to apply a pure shear strain to the unit cell.


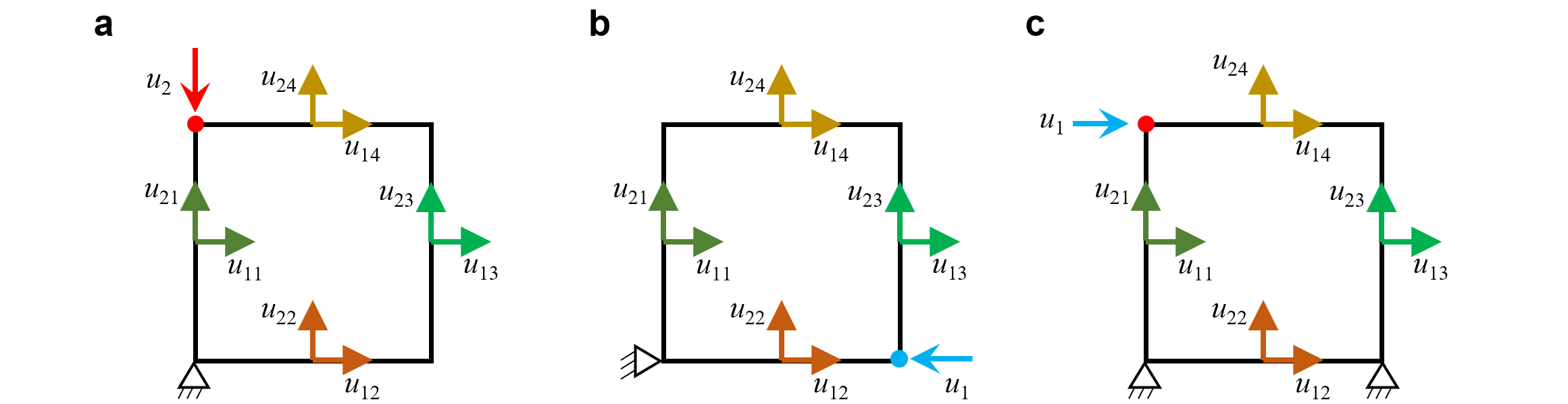


**Supplementary Fig. 4: Periodic boundary conditions. a**, Periodic boundary conditions for calculations of *S*_22_ and *S*_12_. **b**, Periodic boundary conditions for calculations of *S*_11_ and *S*_21_. **c**, Periodic boundary conditions for calculations of *C*_33_ and *C*_23_.

**Supplementary Note 5**

**Representative volume element**

The representative volume element method is used to calculate the effective elastic tensors. With an average strain ($\bar{\varepsilon}_{xx}$, $\bar{\varepsilon}_{yy}$, or $\bar{\gamma}_{xy}$) applied to the unit cell, the effective elastic tensors can be calculated using the corresponding average stress ($\bar{\sigma}_{xx}$, $\bar{\sigma}_{yy}$, or $\bar{\sigma}_{xy}$), which are given as

where *M* is the area of the solid in a unit cell and *a* is the side length of the square unit cell.

**Supplementary Note 6**

**The effect of hyperelastic in nonreciprocity**

We change hyperelastic material into linear material with constant elastic modulus, and then evaluate the constitutive asymmetry *r* in consideration of geometrical and contact nonlinearity as shown in Supplementary Fig. 5. It can be observed that the constitutive asymmetry *r* from hyperelastic material is close to that from linear material. Thus, we confirm that the hyperelastic nonlinearity has negligible influence on the nonreciprocal behavior.


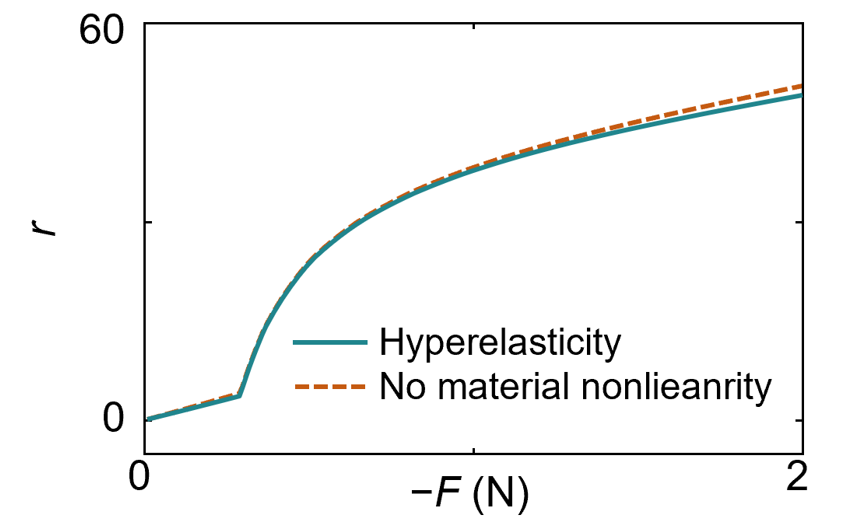


**Supplementary Fig. 5: Comparison between hyperelastic material and linear material.**

**Supplementary Note 7**

**Experiments**

For the orthogonal mode and reconfigurable strategy of cuts, in consideration of the large Poisson’s ratios of the metamaterials, rolling supports are used for the samples in the tests of longitudinal compressions as shown in Supplementary Fig. 6a, d, which are used to provide sliding boundary conditions. The input and output displacements are obtained by tracking the boundary points of the sample. In the reconfigurable strategy of cuts, external limiters are used to restrict the cut, which changes the instruction '0' to instruction '1' as shown in Supplementary Fig. 6d. The sample standard deviations of the experiments for reprogrammable nonreciprocity by the reconfigurable strategy are presented in Supplementary Fig. 8. In Supplementary Fig. 9, we present a framework that provides sliding boundary conditions based on guide rails and sliders, which is used in the experiments of the mechanical diode in Fig. 5a.


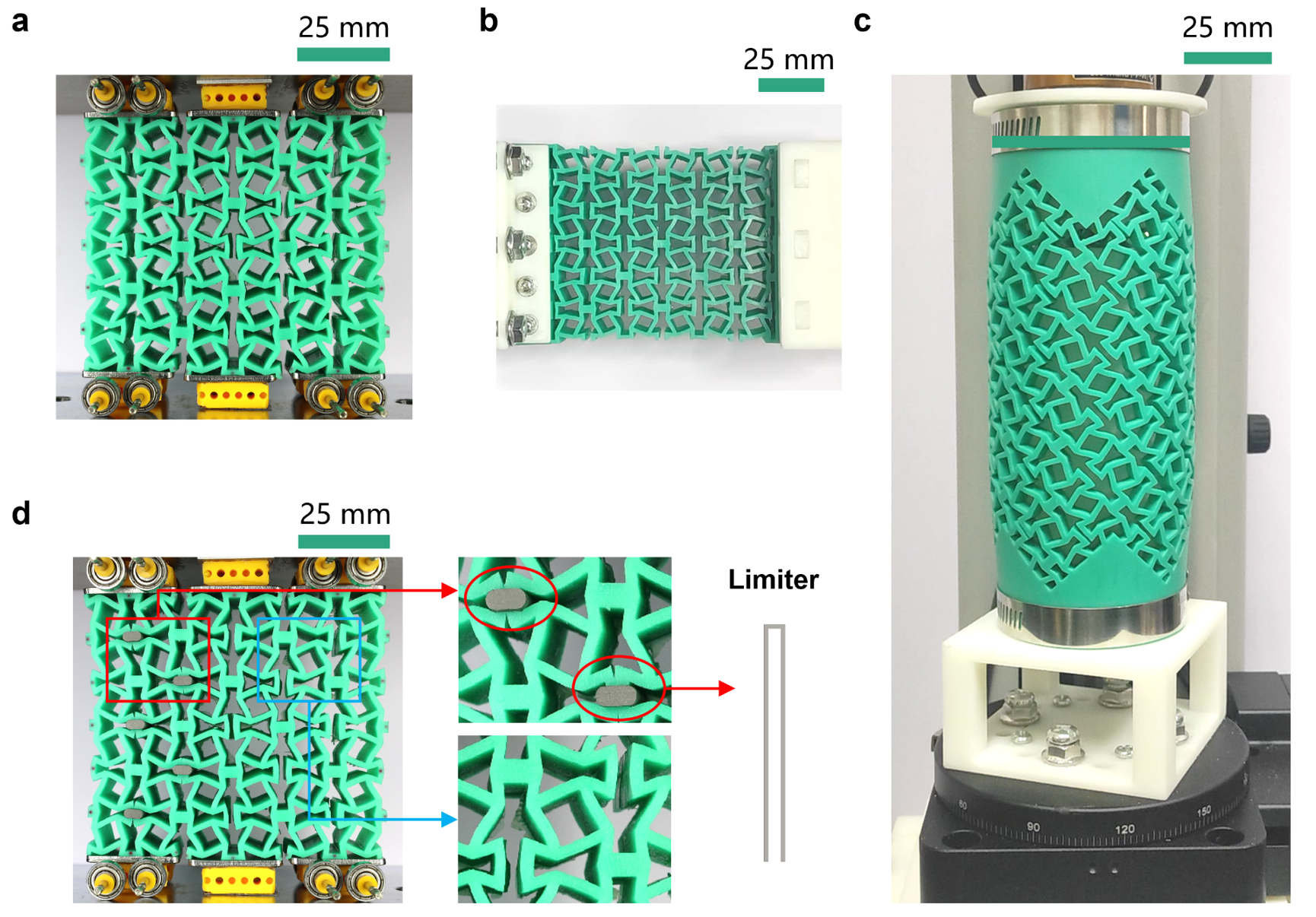


**Supplementary Fig. 6: Experiments. a**, Compression test of the orthogonal mode. **b**, Tension test of the uniaxial mode. **c**, Torsion test of the shear mode. **d**, External limiters used in the reconfigurable strategy of cuts.

The constitutive tensors *S*_12_, *S*_21_, and *S*_22_ can be calculated by Poisson’s ratios and elastic moduli, which are transformed into dimensionless values by the linear elastic modulus *E*_0_ of the metamaterial’s primary material. In the calculation of Poisson’s ratios, the input and output strains are obtained by tracking the boundary points of the samples. The elastic moduli are evaluated via input force, sectional area, and the input strain. The *C*_33_ and *C*_23_ in planes are calculated via shear force and top reaction force as shown in Eqs. and and Supplementary Fig. 7, where *t* is the thickness of the metamaterials. When the shear behavior is mapped onto a rotating tube, *C*_33_ and *C*_23_ are approximately calculated by Eqs. and .

**
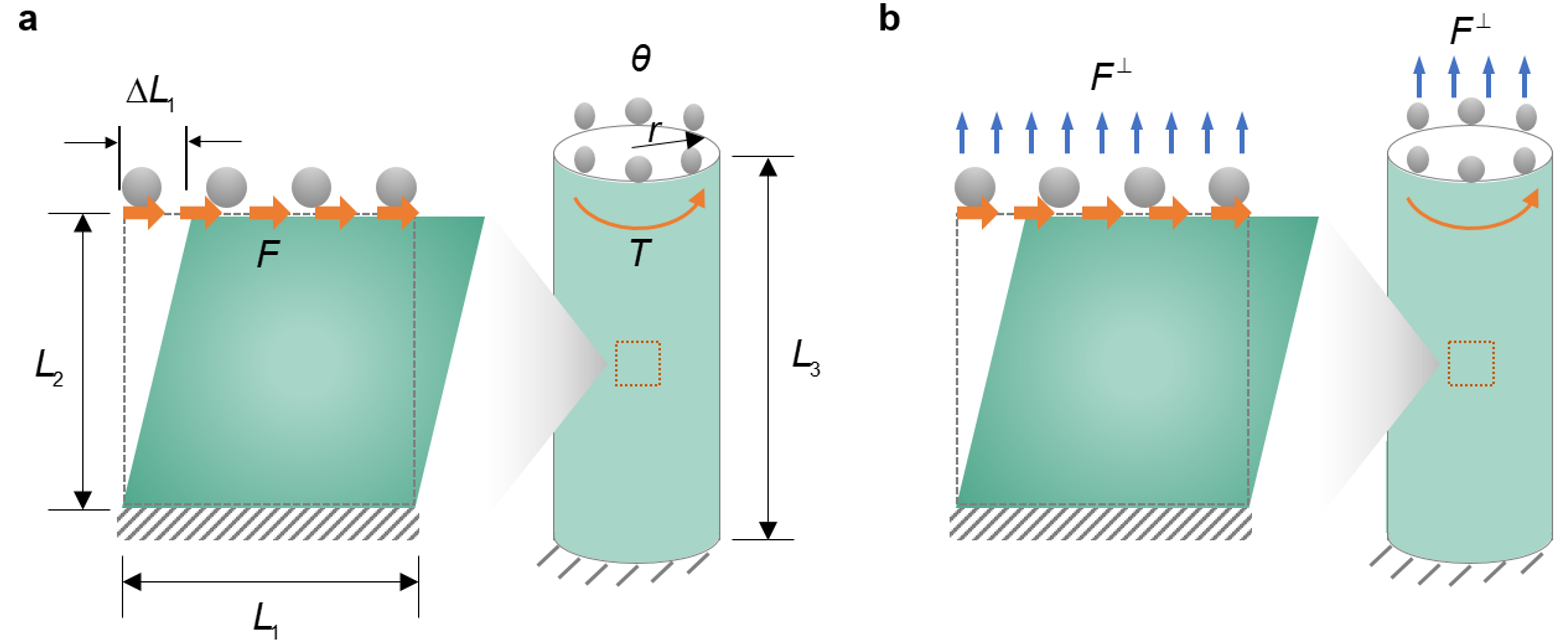
**

**Supplementary Fig. 7: Parameters for calculations of constitutive tensors in tests of shear modes. a**, Torsion angle and torque. **b**, Poynting effect.


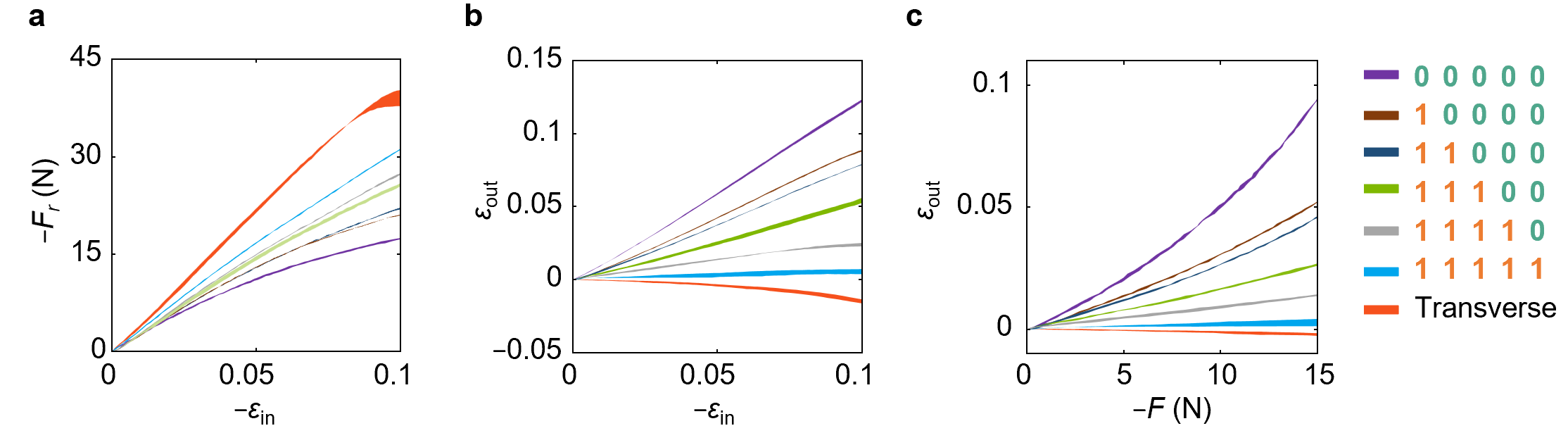


**Supplementary Fig. 8: Data of the experiments for reprogrammable nonreciprocity.** **a**, Reprogrammable reaction force by encoding different sets of binary instructions onto the metamaterial. The boundaries and centers of the color bands are the sample standard deviations and mean values, respectively, which are obtained from five repeated tests. *F* is the compressive force. $\varepsilon_{\text{in}}=u_{\text{in}}/L$. *u*_in_ is the input displacement in the compressive direction. *L* is the side length of the square sample. **b**, Reprogrammable transmission of the displacement field. $\varepsilon_{\text{out}}=u_{\text{out}}/L$. *u*_out_ is the output displacement in the direction that is perpendicular to the compressive force. **c**, Reprogrammable static nonreciprocity. In experiments, the binary instructions are listed in the legends with different markers, all of which are built for longitudinal compression. The responses of experiments under transverse compression are described as 'Transverse' in the legends.


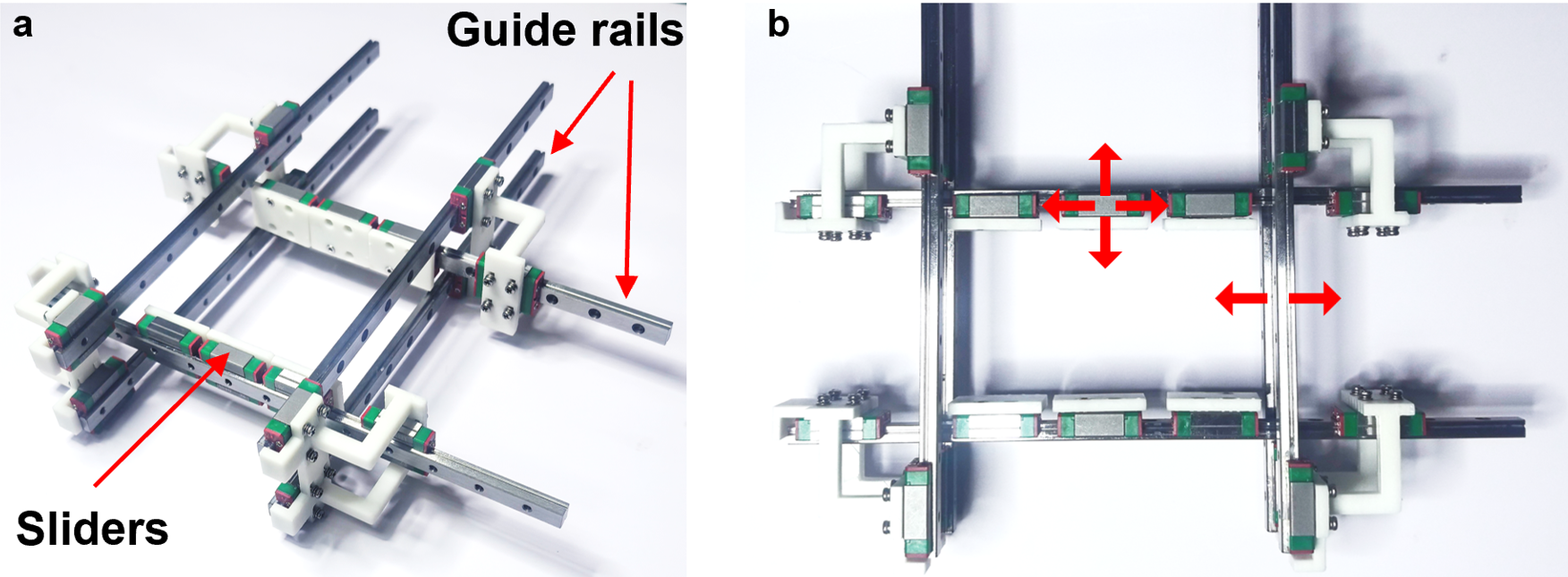


**Supplementary Fig. 9: Framework that** **provides sliding boundary conditions. a**, Guide rails and sliders. **b**, Movement directions.

**Supplementary Note 8**

**Boundary conditions of metamaterials**

For the uniaxial mode, fixed boundary conditions are used as shown in Fig. 2c and d in simulations and experiments, where the left side of the sample is fixed and forces are applied to the right side. Thus, no rolling support is used for the uniaxial mode.

For the shear modes, the bottom of the sample is fixed as shown in Fig. 3a, b and e. The rolling support on the top of the sample is used to apply torque, but it is not necessary. If the top of the sample is also fixed, the experimental method can be changed as shown in Supplementary Fig. 10, where the original sample is extended by mirror symmetry and a rod is used to drive the rotation of the sample at the plane of mirror symmetry. Thus, for the shear modes, rolling support is not necessary. The two ends of the tube can be fixed, and then a torque is applied to drive the rotation of the tube.

**
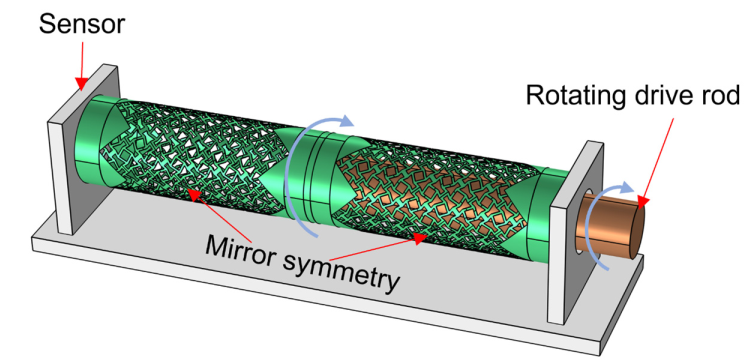
**

**Supplementary Fig. 10:**  **Experimental schematic diagram for samples with fixed ends.**

For the orthogonal mode, rolling supports are considered in the schematic diagram of the nonreciprocal system as shown in Supplementary Fig. 11a. Under these boundary conditions, the deformation modes can be described by elastic moduli and Poisson’s ratios (Supplementary Note 2). If the left and bottom of the sample are fixed as shown in Supplementary Fig. 11b, the deformation modes are different from those considered in this study. In addition, as shown in Supplementary Fig. 11c, the boundary conditions are changed under different loads, which breaks the invariance of the system and should be avoided during the analysis of nonreciprocity. Thus, if the left and bottom of the sample are fixed, the boundary conditions will have a significant impact on the result, because they significantly change the framework of the system with orthogonal nonreciprocal modes and make the system deviate from the orthogonal deformation modes considered in this study. In Supplementary Fig. 11d, we show that four sides with rolling supports can be simplified to two sides for each load. In experiments and simulations, we use the simplified boundary conditions as shown in Fig. 2b.


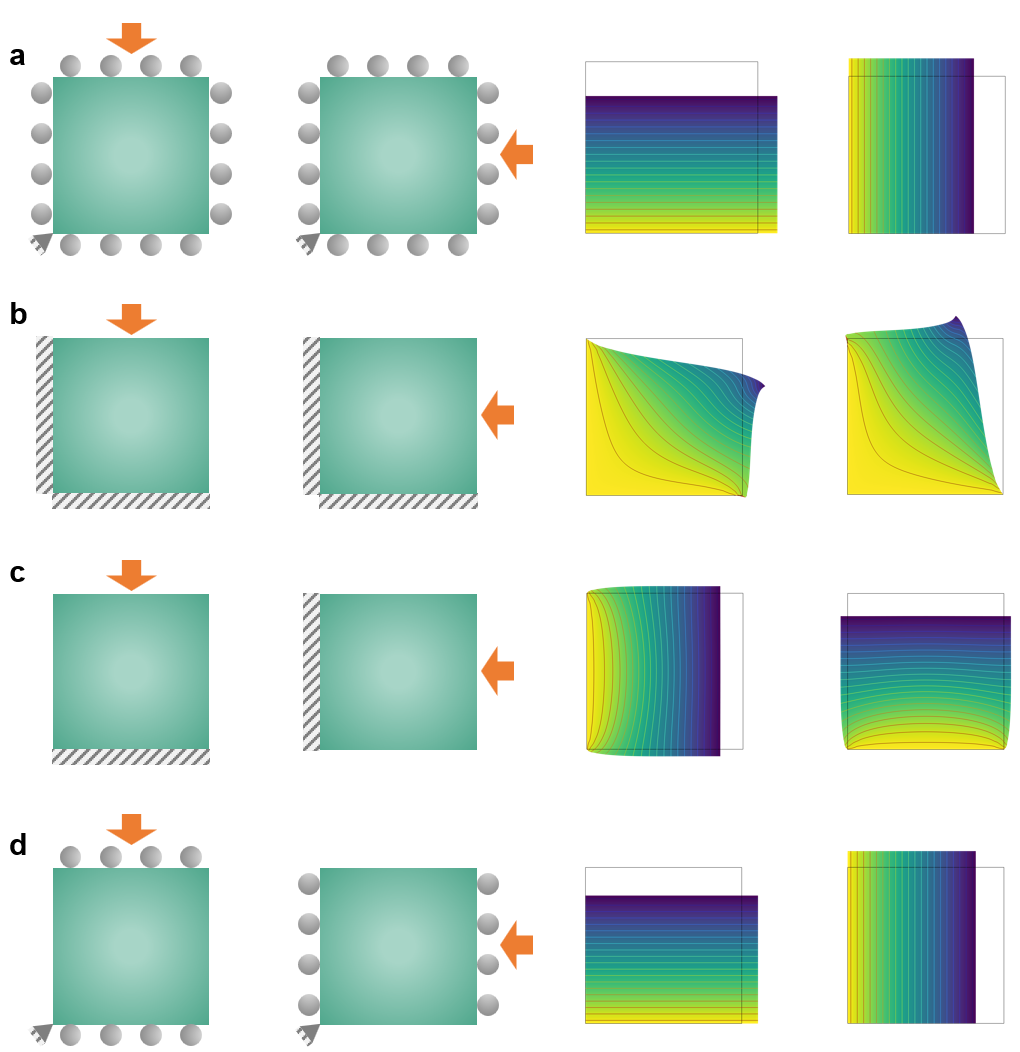


**Supplementary Fig. 11:**  **Simulations under different boundary conditions (a-d).**

If the boundary conditions have not changed radically, the metamaterials in experiments can well overcome the small friction in rolling supports (compared with simulations of structures) and imperfect periodic boundary conditions (compared with simulations of unit cells by the method of representative volume element), as shown in Fig. 2e-h.

**Supplementary Note 9**

**Potential applications in soft robots**

The tube with nonreciprocal metamaterial in Supplementary Fig. 12 shows a soft state under tension and a hard state under compression, where the compression modulus is ten times the tensile modulus and the difference is tunable in situ via reconfigurable cuts. This polarized stiffness allows the metamaterial to act as an exoskeleton for the octopus-like soft robots. The hard mode can improve the structural stiffness of the tentacles so that the robots can stand under a heavy load. The soft mode allows the tentacles to extend so that the robots do not need to overcome high structural stiffness during locomotion, as shown in Supplementary Fig. 12. Thus, the designed metamaterials have the potential applications in auxiliary exoskeletons outside the soft robot bodies. For multi-directional flexibility, as shown in Supplementary Fig. 12, the metamaterial auxiliary exoskeletons can allow the soft robot bodies to elongate and bend.


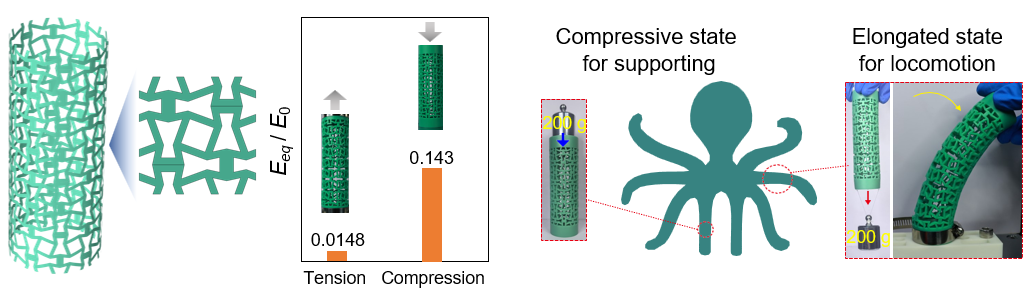


**Supplementary Fig. 12:** **Ectoskeleton for soft robots and effective elastic moduli evaluated by experiments.** The parameters of the microstructure are *w*_1_ = 0.06*a*, *w*_2_ = 0.2*a*, *b* = 0.28*a*, and *θ* = *π*/9. *w*_3_ = 0.0002*a*. The tube includes 4×4 cells. The thickness of the tube is 5 mm.
